# Transcriptome-Wide Identification of the GRAS Transcription Factor Family in *Pinus massoniana* and Its Role in Regulating Development and Stress Response

**DOI:** 10.3390/ijms241310690

**Published:** 2023-06-27

**Authors:** Ye Yang, Romaric Hippolyte Agassin, Kongshu Ji

**Affiliations:** State Key Laboratory of Tree Genetics and Breeding, Co-Innovation Center for Sustainable Forestry in Southern China, Nanjing Forestry University, Nanjing 210037, China

**Keywords:** *Pinus massoniana*, GRAS, abiotic stresses, hormone treatments, development, expression

## Abstract

*Pinus massoniana* is a species used in afforestation and has high economic, ecological, and therapeutic significance. *P. massoniana* experiences a variety of biotic and abiotic stresses, and thus presents a suitable model for studying how woody plants respond to such stress. Numerous families of transcription factors are involved in the research of stress resistance, with the GRAS family playing a significant role in plant development and stress response. Though *GRAS*s have been well explored in various plant species, much research remains to be undertaken on the *GRAS* family in *P. massoniana*. In this study, 21 PmGRASs were identified in the *P. massoniana* transcriptome. *P. massoniana* and *Arabidopsis thaliana* phylogenetic analyses revealed that the PmGRAS family can be separated into nine subfamilies. The results of qRT-PCR and transcriptome analyses under various stress and hormone treatments reveal that PmGRASs, particularly PmGRAS9, PmGRAS10 and PmGRAS17, may be crucial for stress resistance. The majority of PmGRASs were significantly expressed in needles and may function at multiple locales and developmental stages, according to tissue-specific expression analyses. Furthermore, the DELLA subfamily members PmGRAS9 and PmGRAS17 were nuclear localization proteins, while PmGRAS9 demonstrated transcriptional activation activity in yeast. The results of this study will help explore the relevant factors regulating the development of *P. massoniana*, improve stress resistance and lay the foundation for further identification of the biological functions of PmGRASs.

## 1. Introduction

In their capacity as regulatory proteins, transcription factors (TFs) can bind to specific DNA sequences (cis-acting elements) that may be found in the promoters of target genes. Throughout the plant life cycle, TFs play a critical role [1] in plant development and response to abiotic stresses such as drought, salinity, low and high temperatures and the presence of cadmium [2].

The GRAS family is derived from gibberellin insensitive (GAI), gal repressor (RGA) and scarecrow (SCR), and its members are thought to be structurally distinct regulatory proteins that are specific to plants [3]. With a range of sequence lengths and amino acid configurations, the protein for GRAS typically has 400 to 800 amino acids and a coding sequence length of 1200–2500 base pairs (bp). The LHR I, VHIID, LHR II, PFYRE, and SAW structural domains make up the c-terminus of the GRAS protein, which has a highly conserved structure, whereas the N-terminus is varied [4]. Among these, the LHR I–VHIID–LHR II complex, crucial for DNA and protein binding [5], is formed by the highly conserved core region VHIID, which is situated between two leucine-rich areas, LHR I and LHR II. Although PFYRE does not exhibit the same degree of tight conservation as the VHIID area, it nevertheless exhibits significant levels of homology with all proteins. Near the C-terminus of practically all GRAS proteins lies the SAW region. GRAS proteins’ N-termini, whose intrinsically disordered regions (IDRs) [6] can bind to various other proteins, are functional elements. There are numerous subfamilies in the GRAS gene family, and their protein sequences share a tremendous amount in common while also differing significantly. Based on early phylogenetic analysis, GRAS proteins in *Arabidopsis thaliana* have been classified into eight subfamilies: DELLA, LAS, SCR, SHR, PAT1, HAM, SCL9, and SCL4/7 [7]. Later, Cenci and Rouard postulated that the GRAS family of angiosperms also comprises NSP 1, NSP 2, DLT (DWARF and LOW TILLERING) [8], and other subfamilies in addition to these eight subfamilies. There are 16 subfamilies of common bean [9], 9 subfamilies of chickpea [10], and 17 subfamilies of rose [11], for instance. Research on the GRAS family has concentrated on gibberellin (GA) signaling, plant development, stress response, flower induction, and root signal transduction pathways [12]. Members of the GRAS family are engaged in various essential processes of plant growth and development. For instance, DELLA proteins, which impede GA signaling, are essential negative regulators in the GRAS family, controlling plant growth [13], root development [14], and stress response [15]. PAT1 reacts to light cues [16], and the binding of SHR and SCR proteins is linked to the creation of radial root patterns [17]—a dwarf phenotype resulting from the ectopic expression of PpeDELLA1 and PpeDELLA2 in *A. thaliana* [18]. *A. thaliana* lateral shoot development and growth are influenced by LAS proteins [19]. The SCR/SHR complex, composed of the proteins SCR and SHR, is essential for determining the direction of plant root expansion [20]. The WUS-CLV3 interaction module, which controls stem cell maintenance and development of stem tip meristematic tissue [21,22], also involves members of the HAM subfamily. GRAS proteins affect how plants react to biotic or abiotic challenges. For instance, transcription factor BrLAS in *Brassica napus* enhances drought tolerance in transgenic Arabidopsis plants by boosting wax production and decreasing stomatal opening in the leaves [23]. GRAS has also been shown to respond to several hormones. For instance, after GA treatment, the expression of genes such as RcGRAS6 in *Rosa chinensis* drastically altered [11]. The transcription of NtGRAS1 was greatly increased by H_2_O_2_ and SA [24]. The weight of a tomato’s fruit was lowered as a result of the downregulation of GRAS2 in the signal transduction and gibberellin production pathways [25]. As a positive transcriptional regulator for abscisic acid (ABA) and other abiotic stressors, VaPAT1 in mountain grapes has this role [26].

The GRAS family has so far been found in various plants. For instance, rice [27], poplar, tomato [28], strawberry [29], and Arabidopsis [4]. However, there are few studies on the GRAS family in gymnosperms. *Pinus massoniana* is a conifer with a vast distribution in China and is economically significant. It is one of China’s most important timber tree species, and, in its use for the manufacturing of chemicals such as rosin and turpentine, it represents a crucial raw resource with significant economic and environmental advantages [30]. *P. massoniana* confronts various unfavorable settings due to the ecological degradation incurred through global warming, and the economic losses incurred via biotic and abiotic pressures are particularly severe [31]. Therefore, it is necessary to research *P. massoniana* resistance. It is commonly recognized that GRAS family members are crucial for plant growth and resistance to stress. Nevertheless, more information about the GRAS family is needed because there is limited genetic data on conifers.

In this study, we screened 21 GRAS TFs in *P. massoniana*. We used bioinformatics techniques to conduct a thorough analysis based on transcriptome data, including physicochemical characteristics, phylogeny, conserved motifs, and the structural characteristics of PmGRAS proteins. Additionally, we addressed the ways in which PmGRASs express themselves in various tissues, under various abiotic conditions, and in response to various hormone treatments. Based on transcriptome expression data and qRT-PCR results, some genes were selected for additional functional analysis. This research lays the groundwork for further thorough investigations into the function of PmGRASs in controlling stress response and developmental regulation.

## 2. Results

### 2.1. Identification and Multiple Sequence Analysis of GRASs in P. massoniana

First, we downloaded 33 GRAS proteins (AtGRAS) from the TAIR website. Fifty-two candidate GRAS family genes were retrieved from the three transcriptome databases using the hidden Markov model (HMM) (PF03514.13). The CD-search tool was then utilized to carry out additional validation based on these gene models to increase accuracy and verify the existence of GRAS structural domains. After removing the duplicated sequences and wrongly predicted GRASs, 21 GRASs were chosen and designated as *P. massoniana* genes. PmGRAS1 through PmGRAS21 were the names of these GRASs (Table S1). The coding sequences of the validated PmGRASs are displayed in the Supplementary Materials.

The sequences were further examined using the online ExPASy ProtParam tool for their physicochemical characteristics. According to the findings, PmGRASs range in size from 316 amino acids (PmGRAS2) to 842 amino acids (PmGRAS6). The molecular weights of the proteins ranged from 34.3 kD (PmGRAS2) to 92.9 kD (PmGRAS6), the isoelectric points (*pI*) from 4.66 (PmGRAS45) to 6.55 (PmGRAS12), the instability coefficients from 39.62 to 59.03, and the aliphatic amino acid indexes from 43.26 to 88.07. The hydrophilic indexes ranged from −0.523 to 0.042 (Table S2). All proteins had predicted nuclear localization with the exceptions of PmGRAS4 and PmGRAS10, which showed localization in chloroplasts, according to the projected results of CELLO and PSORT for the subcellular localization of PmGRAS1-PmGRAS21 proteins (Table S2).

### 2.2. Phylogenetic Analysis of GRAS Protein Sequences in P. massoniana and A. thaliana

Based on the identified 21 PmGRASs of *P. massoniana*, 33 AtGRASs of *A. thaliana*, 106 PtGRASs of *Populus trichocarpa* and 50 OsGRASs of *Oryza sativa*, a phylogenetic tree with a bootstrap value of 1000 was constructed using the neighbor joining method (NJ) in MEGA7. The coding sequences are available in Supplementary Materials Table S3 and Table S4, the latter of which contains a list of each of these sequential entry numbers.

**Figure 1:**
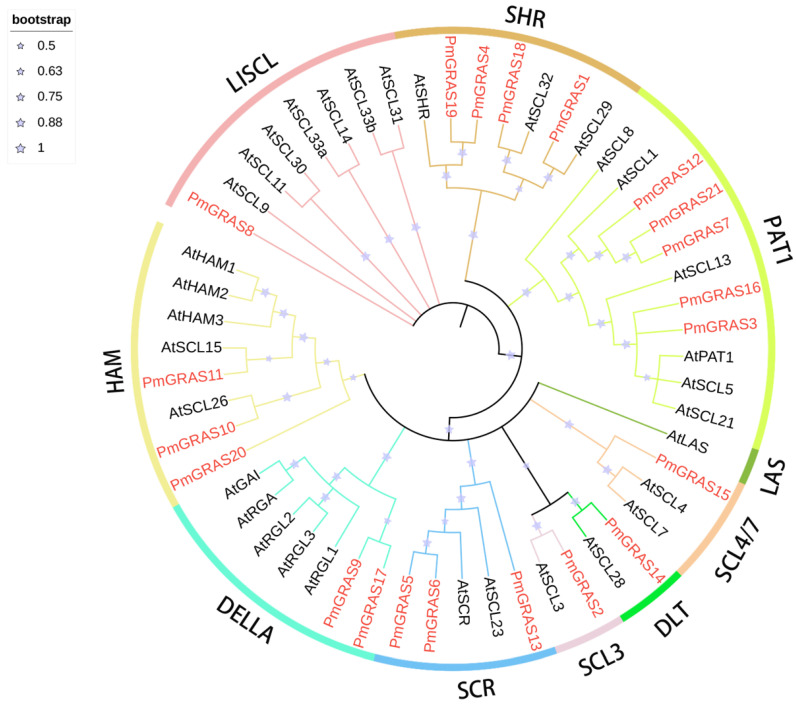
Phylogenetic grouping of members of the GRAS family of *P. massoniana.* Different colored rings denote various subfamilies, while red letters denote *P. massoniana* GRAS proteins.

First, we created a phylogenetic tree using 33 AtGRAS proteins from *A. thaliana* and 21 PmGRAS proteins from *P. massoniana*. Following earlier research on *A. thaliana* (Figure 1), the clustering results showed that the 54 GRAS proteins were classified into 10 subfamilies, of which a total of 9 (SCL3, HAM, DELLA, SCR, LAS, LISCL, SHR, PAT1 and SCL4/7) contained *P. massoniana* GRAS proteins. PAT1, which had a total of five members, and SHR, which had four members, were the two groups with the most PmGRAS proteins. According to the bootstrap test, several PmGRAS proteins clustered well with AtGRAS proteins. These proteins might have physiological properties in common with AtGRASs. Additionally, the analysis outcomes above were supported by the phylogenetic tree of GRAS proteins from *P. massoniana* constructed using the same methodology (Figure S1).

We also created phylogenetic trees for four species of *P. massoniana* (a gymnospermia), *A. thaliana* (a eudicots plant), *Populus* (a woody plant), and *O. sativa* (a monocots plant) in order to research the phylogenetic history of the GRASs family (Figure S1). The 210 GRAS proteins were classified into 13 subfamilies, most of which were supported by good posterior probabilities (>0.9) and guidance values (>60%). AtSHR, AtPAT1, AtSCR, AtSCL4/7, AtLAS, Os19, SCL3, HAM, Os4, Pt20, DLT, AtSCl3, DELLA, and LISCL were the 14 subfamilies. In general, most subfamilies had GRAS members from four species. However, the absence of genes from *P. massoniana* and *A. thaliana* in the Os4 and Os19 subfamilies suggests that these two plant species have lineage-specific gene deletions. PmGRAS20 of the HAM subfamily was clustered into the Pt20 poplar-specific subfamily [32] in the clustering map of the four species, establishing a strong monophyletic branch.

### 2.3. Analysis of PmGRAS Family Protein Motifs and Gene Structures

**Figure 2:**
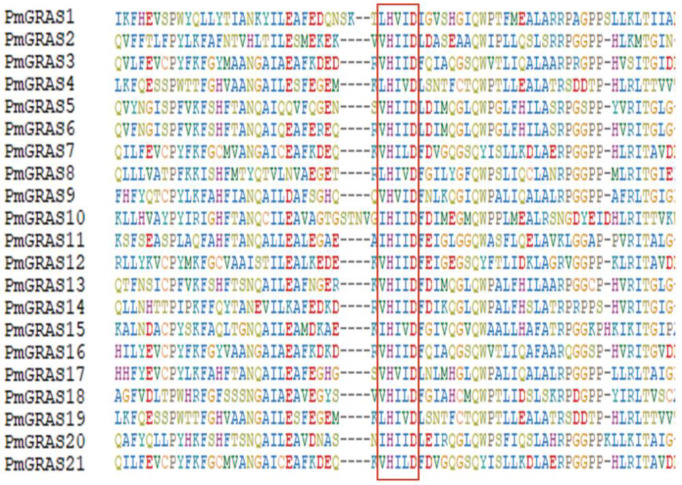
VHIID domain of 21 PmGRAS proteins based on sequence alignment. The most conserved GRAS domain of VHIID is boxed in red.

**Figure 3:**
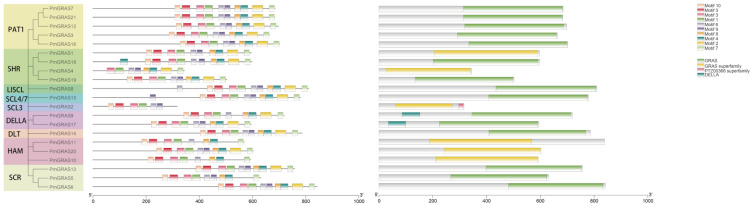
Phylogenetic grouping, motif distribution and structural domain analysis of GRAS proteins in *P. massoniana*.

A peptide sequence comparison was made to examine *P. massoniana*’s GRAS structural domain. All 21 PmGRAS proteins included VHIID (Figure 2), LHRI, and 19 also contained SAW structure domains, with individual genes containing incomplete LHRI structural domains. The absence of the SAW structural domain and the presence of only a tiny fraction of all structural domains except the VHIID structural domain in the *PmGRAS2* protein raises the possibility that this protein serves a purpose distinct from other PmGRAS proteins. The DELLA subfamily contains the DELLA domain, another conserved structure (Figure 3). Over a dozen direct DELLA TFs have been reported, indicating the diverse functions of DELLA in plants. [33].

The conserved motifs of 21 PmGRAS protein sequences were discovered using MEME software (Figure 3), and 10 different conserved motifs (called motifs 1–10) were revealed. The 10 conserved motifs are listed in Table S5 and illustrated in Figure S2. All PmGRAS members shared these motifs, which were spread among the five domains—LHR I, VHIID, LHR II, PFYRE, and SAW. We discovered that all PmGRAS proteins contain motifs 1, 3, and 6, which, combined with the sequence comparison results, suggests that these motifs play significant roles in the GRAS family. The distribution and features of reverse motifs in the same GRAS subfamily were similar and consistent with the phylogenetic tree’s categorization results. This suggests that the functions and conservation of the genes within the same subfamily are comparable. Motifs 10, 5, 3, 1, 6, 8, 4, 2, and 7 can be found, for instance, in the DELLA subfamily and the PAT subfamily. Some motifs were frequently distributed at particular locations within the pattern; for example, motif 10 is frequently distributed near the pattern’s start, whereas motif 7 is virtually always distributed at the pattern’s conclusion. Most of these preserved motif roles are still yet to be fully understood. When considered collectively, the variability in motif quantity and composition among GRAS subfamilies raises the idea that these genes may have served various purposes during evolution due to variations in the distribution of gene motifs.

### 2.4. Subcellular Localization Analysis of PmGRASs

**Figure 4:**
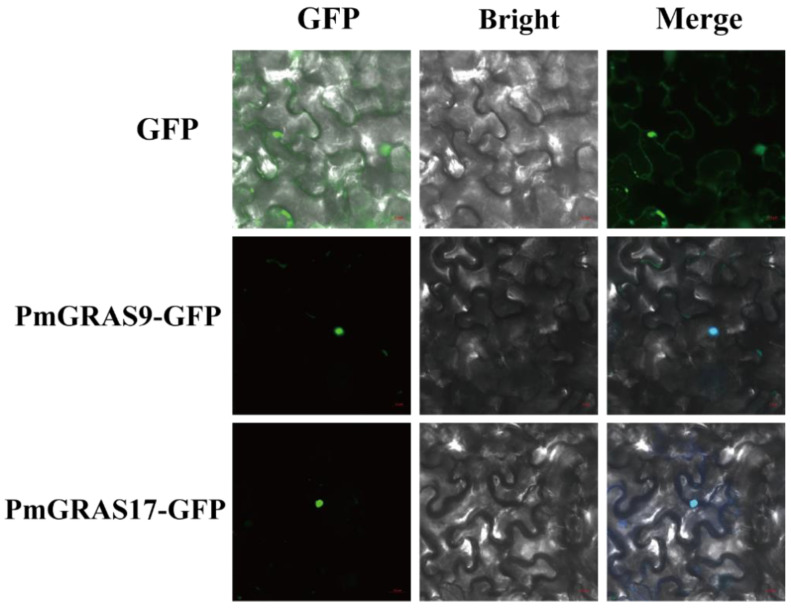
Subcellular localization analysis of the PmGRAS9 and PmGRAS17 proteins. Transient expression of GFP (control), PmGRAS9-GFP and PmGRAS17-GFP in *Nicotiana benthamiana* leaves. The scale in the images of GFP and PmGRAS9-GFP is 10 µM, and the scale in the images of PmGRAS17-GFP is 20 µM.

We chose the DELLA subfamily proteins PmGRAS9 and PmGRAS17, which have been shown to have various functions in other species [34]. We created transient expression vectors by fusing GFP into the ORF to validate the results anticipated from the subcellular localization site. Laser confocal scanning microscopy revealed a fluorescent signal in the nucleus (Figure 4), which was in line with the expected outcomes. Therefore, the gene’s location in the nucleus can be determined by comparison with the fluorescence map of the empty load.

### 2.5. Transcriptome Analysis of Needles of P. massoniana under Drought Stress

**Figure 5:**
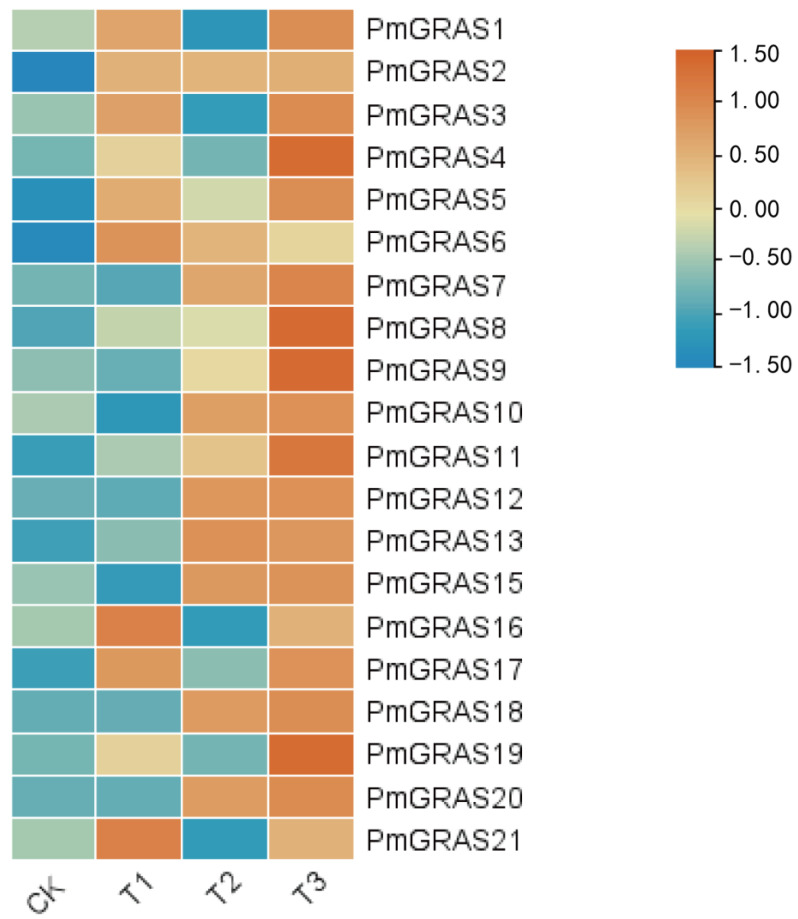
GRAS family members’ transcriptomic profiles in *P. massoniana* at various drought levels: CK (80 ± 5)%, T1 (65 ± 5)%, T2 (50 ± 5)% and T3 (35 ± 5)%. Heat maps were created by performing a row scale on log2 (FPKM + 0.01) values, where the color scale denotes relative expression levels.

Based on transcriptome information on drought stress, a heat map of 20 PmGRASs was created (Figure 5). Due to its low expression, PmGRAS14 might not be found in the transcriptome. The heat map displayed the levels of the expression of these 20 genes under various levels of drought stress. Compared with the CK group, the expression of all genes was significantly upregulated under drought treatment. Among these, the expression of six genes—PmGRAS2, PmGRAS8, PmGRAS11, PmGRAS13, PmGRAS18, and PmGRAS20 showed a consistent rise in response to drought. Under mild conditions, the expression of five genes—PmGRAS7, PmGRAS9, PmGRAS10, PmGRAS12, and PmGRAS15 showed a constant increase. Nine genes—PmGRAS1, PmGRAS3, PmGRAS4, PmGRAS5, PmGRAS6, PmGRAS16, PmGRAS17, PmGRAS19, and PmGRAS21 had their expression rise following a mild drought treatment before falling under a moderate drought condition. The majority of PmGRASs were generally responsive to drought treatment and expressed positively. These findings suggest that PmGRAS is critical for drought stress resistance.

### 2.6. Expression Pattern of PmGRASs under PEG and Mechanical Injury Stress

**Figure 6:**
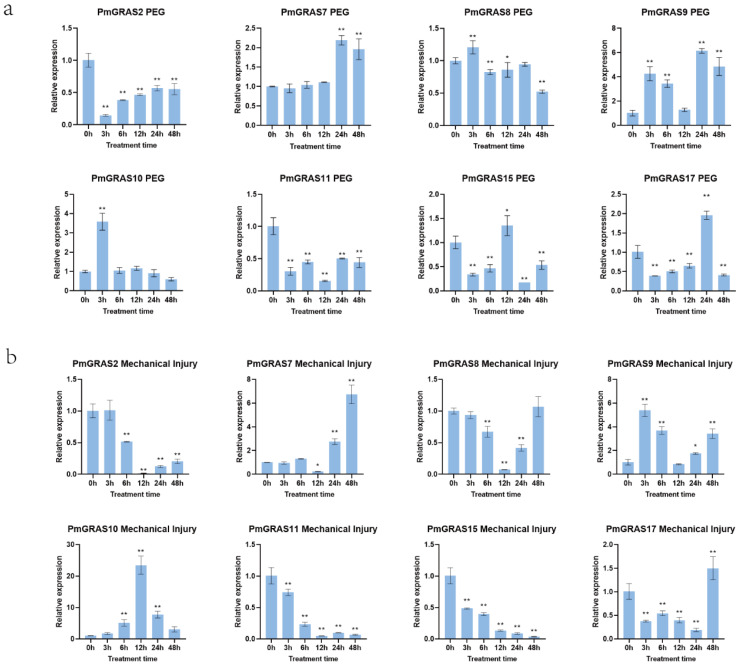
Expression of PmGRASs under different stresses. (**a**) PEG and (**b**) mechanical injury. The relative expression level is indicated as the mean ± standard deviation (SD), asterisks indicate significant differences in transcript abundance in the treated group compared with the control group (0 h) (student’s *t*-test, * *p* < 0.05, ** *p* < 0.01).

We chose eight PmGRASs for RT-PCR expression analysis—PmGRAS2, PmGRAS7, PmGRAS8, PmGRAS9, PmGRAS10, PmGRAS11, PmGRAS15, and PmGRAS17—to see how they responded under different stress treatments. These genes were chosen based on some highly expressed genes in the drought stress transcriptome expression data and on their identification in other species associated with the regulation of development and stress response [35]. The results show how these eight genes expressed under mechanical injury and PEG stress (Figure 6a). Some genes clearly responded when subjected to PEG treatment. PmGRAS2 and PmGRAS11 expression fluctuated between decline and rise, while PmGRAS7 and PmGRAS17 expression significantly increased after 24 h. PmGRAS10 expression increased, dropped, and then increased significantly after three hours. Neither PmGRAS8 nor PmGRAS15 responded to PEG stress. PmGRAS9 showed a substantial increase in expression in response to PEG administration at 3 h, 6 h, 24 h, and 48 h when compared with 0 h. All genes responded significantly to the treatment for mechanical injury (Figure 6b). While the expressions of PmGRAS11 and PmGRAS15 dramatically reduced, those of PmGRAS2, PmGRAS8, and PmGRAS17 all roughly showed a lowering and then increasing pattern. At 12 h, the expressions of PmGRAS2, PmGRAS7, PmGRAS8, and PmGRAS11 were very low. At 24 and 48 h, PmGRAS7 expression considerably increased, increasing 2.8 and 6.7 fold, respectively, over 0 h. PmGRAS9’s expression likewise saw a considerable alteration, spiking quickly and then falling. The expression of PmGRAS10 dramatically changed in response to the mechanical injury stress, first rising sharply and then falling. At 12 h, it peaked at 23.5 times higher than 0 h.

### 2.7. Expression Pattern of PmGRASs under Different Hormone Treatments

**Figure 7:**
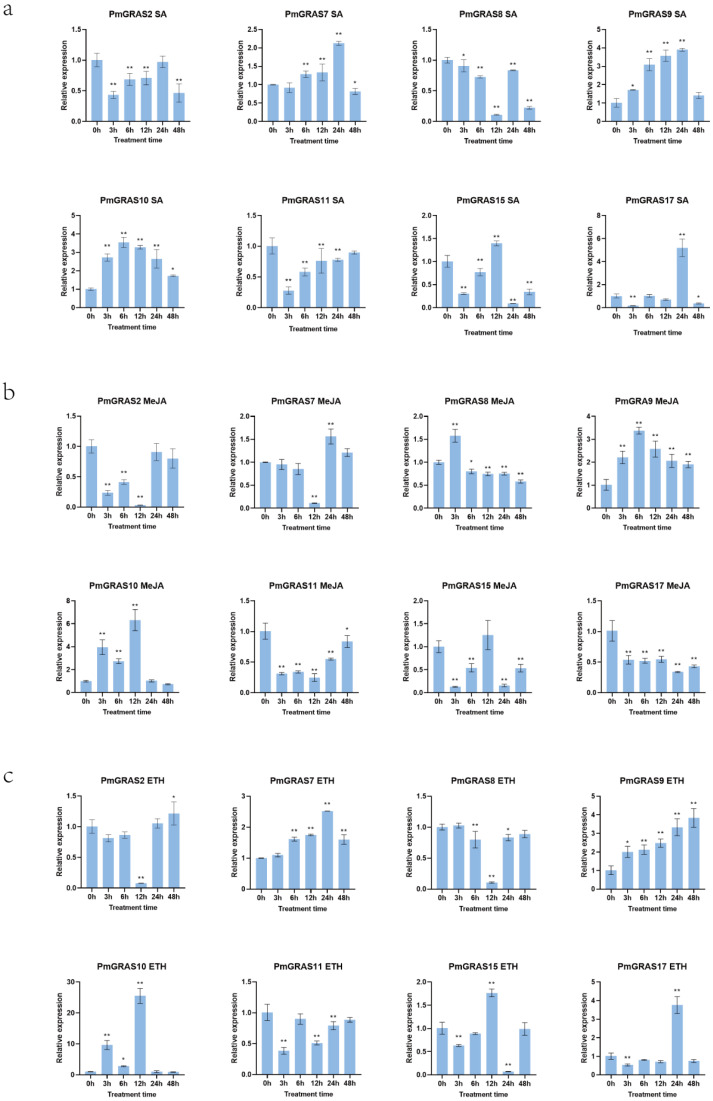

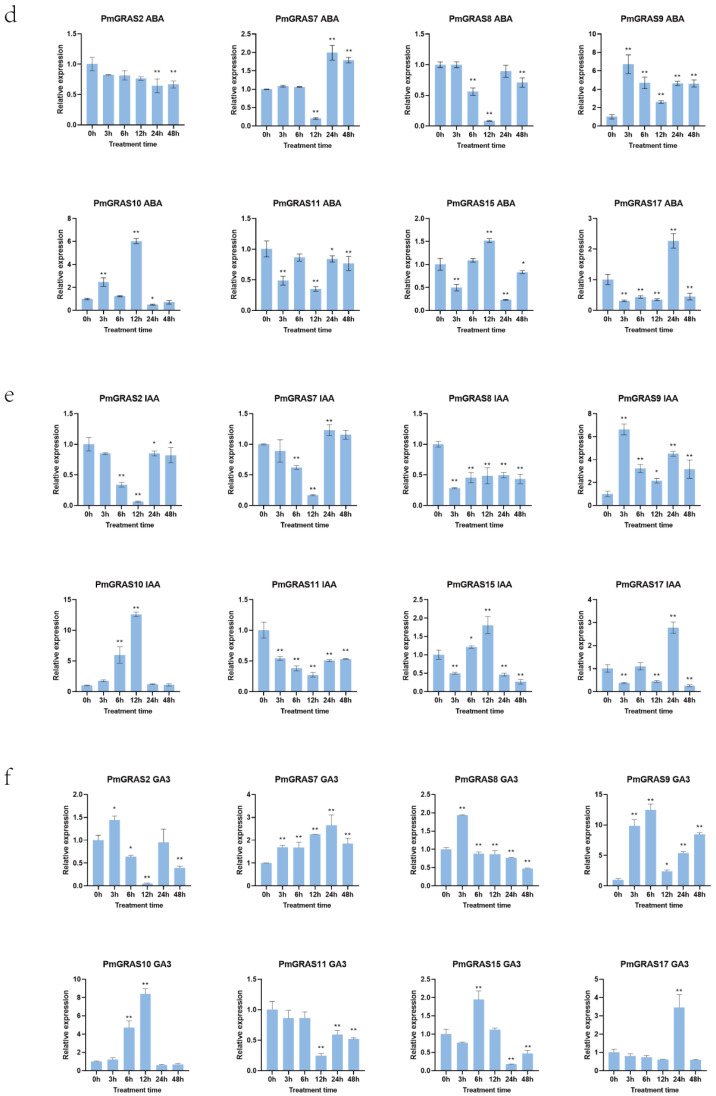
Expression profiles of PmGRASs under different hormone treatments. (**a**) SA, (**b**) MeJA, (**c**) ETH, (**d**) ABA, (**e**) IAA, and (**f**) GA3. The relative expression levels are indicated as the mean ± standard deviation (SD), asterisks indicate significant differences in transcript abundance in the treated group compared with the control group (0 h) (student’s *t*-test, * *p* < 0.05, ** *p* < 0.01).

After this, we exposed eight PmGRASs to varying hormone treatments, and the expression patterns of these genes under distinct hormone treatments are showcased in Figure 7. PmGRAS7, PmGRAS9, and PmGRAS10 expression increased and then dropped in response to SA treatment (Figure 7a), with PmGRAS7 and PmGRAS9 reaching the greatest expression levels at 24 h and PmGRAS10 at 6 h. SA did not effect PmGRAS2, and PmGRAS11 expression fluctuated back and forth. PmGRAS8 expression peaked at 24 h, 5.2 times higher than at 0 h, while PmGRAS17 expression peaked at 12 h. Under MeJA treatment (Figure 7b), the expression of PmGRAS2, PmGRAS12, PmGRAS15, and PmGRAS17 was unstable and did not significantly respond to MeJA. The expression of PmGRAS7 and PmGRAS11 increased and then reduced, while PmGRAS8 and PmGRAS9 expression grew and then declined. At 12 h, PmGRAS10 expression was 6.3 times higher than at 0 h. Most of the genes responded significantly to ETH treatment (Figure 7c). PmGRAS2 and PmGRAS11 were not ETH-sensitive. PmGRAS7 expression increased and subsequently declined, while PmGRAS8 expression decreased and then increased. PmGRAS9 expression grew steadily, reaching a peak at 48 h that was 3.8 times higher than 0 h. At 24 h, the expression of PmGRAS15 was at its lowest, and the expression of PmGRAS17 was at its highest. After reaching its lowest point at 12 h, PmGRAS7 and PmGRAS8 expression rose after ABA treatment (Figure 7d). The highest expression of PmGRAS9 was at 3 h, and was 6.7 times higher than that at 0 h; the highest expression of PmGRAS10 was at 12 h, 6 times higher than that at 0 h; and the highest expression of PmGRAS17 was at 24 h, 2.3 times higher than that at 0 h. PmGRAS2 and PmGRAS11 showed no ABA sensitivity. At 24 h, PmGRAS15 expression was at its lowest. Eight genes responded to IAA to varying degrees (Figure 7e), although PmGRAS2, PmGRAS7, PmGRAS8, and PmGRAS11 had the lowest expression levels at 12 h, 12 h, 3 h, and 12 h, respectively. The most significant expression level of PmGRAS10 was 12.6 times that of 0 h, and it initially climbed and subsequently declined. PmGRAS9 expression peaked at 3 h, when it was 6.6 times higher than it was at 0 h. PmGRAS15 and PmGRAS17 had the highest expression levels at 12 and 24 h, respectively. PmGRAS2 expression under GA3 treatment was lowest at 12 h (Figure 7f), whereas *PmGRAS7*, *PmGRAS8*, and *PmGRAS10* expression increased and then fell, with PmGRAS10 expression 16.8 times higher at 12 h than at 0 h. PmGRAS9 expression reached its maximum at 6 h and was 9.1 times higher than at 0 h. PmGRAS11 and PmGRAS17 expression dramatically increased at 24 h and were 3.5 times greater than at 0 h.

### 2.8. Expression Levels of PmGRASs in Different Tissues

**Figure 8:**
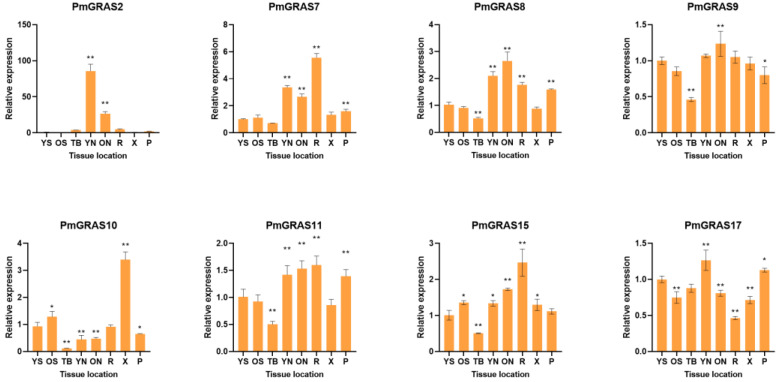
Relative expressions of PmGRASs in eight representative tissues. YS: young stem; OS: old stem; TB: terminal bud; YN: young needle; ON: old needle; R: root; X: xylem; P: phloem. The relative expression levels are indicated as the mean ± standard deviation (SD), asterisks indicate significant differences in transcript abundance in the treated group compared to the control group (0 h) (student’s *t*-test, * *p* < 0.05, ** *p* < 0.01).

Eight PmGRASs were expressed differently in each of the eight tissues represented by the qRT-PCR data in Figure 8: young stem (YS), old stem (OS), terminal bud (TB), young needle (YN), old needle (ON), root (R), xylem (X), and phloem (P). According to the findings, seven genes were found to be expressed in all eight tissues. The expression of PmGRAS2 was about 30 times higher in young needles than it was in old stems, xylem, phloem, and terminal bud. PmGRAS2 was almost completely missing in old stems. It is hypothesized that PmGRAS2 may be crucial for the growth of immature needles. Young needles exhibited the highest levels of PmGRAS17 expression, although these levels did not significantly differ from those in other sections. The expression of PmGRAS8 and PmGRAS9 was highest in the aged needles. Among these, the expression of PmGRAS8 was 2.6 and 2.0 times greater in old needles and young needles, respectively, compared with young stems. Finally, the expression level of PmGRAS9 in terminal bud was 0.46 times lower than that observed in young stems. The most strongly expressed PmGRASs in roots were PmGRAS7, PmGRAS11, and PmGRAS15. PmGRAS7 expression was about 5.5 times higher in roots than in young stems, and it was about 3.3, 2.6, and 1.5 times higher in young needles, old needles, roots, and phloem, respectively. PmGRAS11 expression was about 1.5–1.6 times higher in young needles, old needles, roots, and phloem than in young stems. PmGRAS10, a member of the HAM subfamily, showed a 3.4-fold higher expression in the xylem than in the young stem. Overall, the eight genes showed various patterns of expression across the plant, but the expression levels were noticeably lower in the terminal bud and generally greater in the needles.

### 2.9. Transcriptional Activity Assays of PmGRAS9

**Figure 9:**
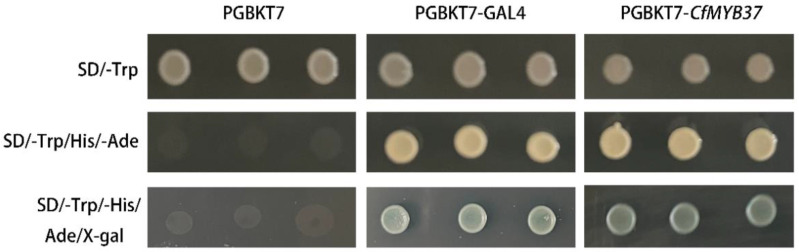
Transcriptional activation assay of PmGRAS9. Empty pGBKT7 vector was used as a negative control.

In addition, among the eight genes we chose PmGRAS9 from the DELLA subfamily to be tested for transcriptional activity (Figure 9). This gene is strongly responsive to non-stress and hormonal treatments. Yeast cells carrying the fusion vector pGBKT7–PmGRAS9 can grow on the SD/-Ade/-His/-Trp-selected medium, and a blue reaction developed with the addition of X-gal. This is depicted in various figures. The findings demonstrate PmGRAS9 to be a transcriptional activator, laying the groundwork for additional research on gene regulation.

## 3. Discussion

As whole genome sequencing has advanced, GRASs have been discovered in numerous plant species as a crucial and diverse set of regulatory molecules that play significant roles in plant development and in responses to a variety of harmful environmental inputs. Increasingly, studies have revealed that GRASs are promising candidates to be the subjects of study in terms of plant development and resistance. However, reports of GRAS families linked to stress in *P. massoniana* are uncommon. As a result, this study offers a thorough analysis of *P. massoniana* GRAS family members and makes educated guesses about the biological roles of some unidentified PmGRAS proteins, particularly in aspects related to stress and growth, providing a useful foundation for further research into the roles of PmGRAS.

### 3.1. PmGRAS Gene Structure and Evolutionary Analysis

Most PmGRAS proteins differ greatly in structure, showing high levels of complexity. GRAS protein sequences are also very varied, with lengths ranging from 316 to 842 amino acids. This variety may be attributed to gene duplication events or chromosomal changes. [36]. The phylogenetic analysis (Figure 1) showed that all *A. thaliana* subfamilies save the LAS subfamily have at least one PmGRAS protein, indicating that these subfamilies have not been lost in long-term evolution. It is possible that some species- or lineage-specific GRAS subfamilies were retained during evolution, given the wide variance in the number of GRAS TF subfamilies among various plant species. Members of the same subfamily share a genetic structure, and members of various subfamilies have biological activities that vary. For instance, DELLA protein is a GA signaling repressor that regulates plant growth and development and is crucial for gibberellin response and plant height regulation [37], among other things. Members of the HAM subfamily have essential roles in the elongation of the petunia flower bud meristem [38], and PtrHAM4-1 overexpression in transgenic *A. thaliana* plants has been shown to significantly increase the development of the vascular formation layer [39]. Current research on the functions of GRAS subfamilies in plant development has primarily centered around the six subfamilies of SHR, SCR, LAS, DELLA, HAM, and SCL. However, further research is necessary to fully understand the roles of other GRAS subfamilies in plant development [35]. It is possible that certain non-conserved GRAS members have vanished during evolution or that some PmGRASs have not been detected due to a lack of genomes, which accounts for the absence of *P. massoniana* members in subfamily LAS. PmGRAS20 was grouped into the HAM subfamily in the phylogenetic trees of two species, whereas it was grouped into the Pt20 subfamily in the phylogenetic trees of four species. The Pt20 subfamily is *Populus*-specific, indicating that it was acquired in the *Populus* lineage following divergence from the most recent common ancestor of *A. thaliana* and *O. sativa*. This subfamily may play a specific role in adaptive evolution of woody vegetation, and similarly new members have successively emerged throughout the evolutionary process. In the future, more studies on GRAS genes in the plant kingdom may identify more subfamilies that can help trace the evolutionary history of addition/deletion or duplication of subfamilies.

The 10 motifs identified in the current investigation were spread among 5 structurally conserved domains: LRHI, VHIID, LRHII, PFYRE, and SAW (Figure S3). Previous research has demonstrated that these motifs can mediate protein–protein and protein–DNA interactions [40]. Members of the same subfamily share a common evolutionary ancestor since GRAS motifs in separate subfamilies are less conservative, whereas they are more conservative within the same subfamily [41]. These conserved motifs are linked to the proteins’ activities and have a common evolutionary history [42]. The PmGRAS protein has a highly disordered region at its N-terminal end, which enables GRAS proteins to function as the major regulators in several signaling pathways, and the more conserved motifs at the c-terminus also vary among different subfamilies of the GRAS protein family. For instance, the DELLA subfamily members PmGRAS9 and PmGRAS17 have distinctive DELLA structural domains at their N-termini (Figure 3), and this inherent structure enables the subfamily to have multiple functions. We also discovered some structural domain losses, such as PmGRAS2 of the SCL subfamily lacking in LHR II and SAW structures. This loss or gain causes gene families to grow [43,44], and this phenomenon has been observed in *O. sativa*, *Zea mays*, and other plants. Additionally, PAT1 has the most preserved structure and a composition of motifs that is identical to *P. massoniana*.

### 3.2. Possible Functions of PmGRAS Members

We studied the transcriptional data of GRASs in *P. massoniana* under drought stress. Additionally, we looked at the expression of eight PmGRASs under various abiotic stresses and hormone treatments, as GRAS members are widely reported to be involved in plant development and stress response. All 20 PmGRASs displayed varying degrees of expression changes in response to drought, and some PmGRASs displayed significant up- or downregulation of expression in response to drought. This suggests that *P. massoniana*’s GRASs are crucial for the plant’s ability to withstand drought stress. Most of the eight genes also displayed notably differential expressions under various treatments.

In earlier studies, the HAM subfamily’s LeHAM3 displayed a high response to salt stress at the seedling stage [45]. In this study, the HAM subfamily’s PmGRAS10 displayed a high response to all treatments, and their expression tended to be highly significant, indicating that they likely play a significant role in *P. massoniana*’s ability to tolerate multiple stresses. In response to GA3 induction, the expression of PmGRAS9 and PmGRAS17, which belong to the DELLA family of genes that respond to gibberellin, increased significantly, indicating that they may have reacted to GA signaling. This is similar to research on GA signaling by AtRGA [46] in *A. thaliana*. Furthermore, PmGRAS9 and PmGRAS17 were implicated in practically all stress responses, consistent with the findings associated with *Setaria italica* [47]. As a result, we surmised that the DELLA-mediated gibberellin signaling pathway plays a significant role in several stress responses in *P. massoniana*. Members of the GRAS family were also discovered to be involved in auxin signaling. Auxin has been shown to alter the expression of AtSCL15 from *A. thaliana* and its homolog, BnSCL1 of *Brassica napus*, both of which have been shown to be strongly expressed in root tissues [4]. The homologs of AtSCL15, PmGRAS10 and PmGRAS11, both demonstrated significant expression changes in our study after 6 h of IAA administration, and PmGRAS11 was likewise highly expressed in the roots, indicating that these genes may share signal transduction roles. Additionally, other subfamily members, such as *PmGRAS9 and PmGRAS15*, responded to IAA treatment, suggesting that the functions are multiple among different subfamilies. The PAT1 subfamily members AtSCL5, AtSCL21, AtSCL13, and AtPAT1 have been shown to be extensively expressed in leaves and implicated in the light signaling pathway [48]. PmGRASs in the PAT1 subfamily may increase resistance to external pressures through these signaling pathways because PmGRAS7, a member of the PAT1 subfamily, has been shown to offer substantial responses to ETH, GA3, and mechanical injury treatments. PeSCL7, a member of the poplar SCL subfamily [49], increased *A. thaliana* seedling’s tolerance to salinity and drought by encouraging root growth and minimizing water loss. Members of the SCL subfamily—PmGRAS2, PmGRAS8, and PmGRAS15—in *P. massoniana* showed steadily increasing trends in expression under drought stress, indicating that these three PmGRASs may also play significant roles in drought resistance in *P. massoniana*. A homolog of AtSCL14, PmGRAS8, demonstrated significant and primarily negative responses to treatments for mechanical injury, SA, GA3, and IAA, which suggests that PmGRAS8 may play a role in stress response as well as in hormone-mediated growth and development pathways. Earlier studies have demonstrated the necessity of AtSCL14 for stress-induced promoter activation [50]. Overall, GRASs are implicated in various stress- and growth-related signaling pathways in plants; however, further functional investigations are required to determine how GRASs precisely react to these signaling pathways.

### 3.3. Role of PmGRASs in the Development of Different Tissues of P. massoniana

The expression of GRAS varies based on the species, stage of development, and environmental factors [51] in many plant organs and tissues, such as the germ, radicle, stem, anther, fruit, primary root, silk, and leaf [52].The majority of the *A. thaliana* SCL3 subfamily members displayed high levels of expression in the roots [53], and its homolog PmGRAS2 was likewise strongly expressed in the roots but at a higher level in the needles, raising the possibility that PmGRAS2 might be involved in photosensitive pigment signaling. Additionally, it was discovered that AtSCL3 was engaged in the GA pathway, SCL3 deletion mutants revealed lower GA response and increased expression of GA biosynthesis genes, demonstrating that SCL3 functions as a positive regulator of the GA signaling system [54]. It was also revealed that light signals could specifically regulate the gibberellin metabolic pathway to promote seedling growth [55]. Thus, we hypothesize that PmGRAS2, which produces a response under gibberellin treatment, also mediates the gibberellin metabolic pathway regulated by light signaling. The PAT1 subfamily member ERF115-PAT1 was discovered to be involved in root meristematic growth [56]. In our study, the PAT1 subfamily member PmGRAS7’s expression pattern was connected with the greatest levels in the roots of *P. massoniana*, indicating that it may be crucial for root development. *A. thaliana* meristem tissues exhibited high levels of expression for the SCL subfamily members AtSCL26 and AtSCL27 [57]. In contrast, vascular meristem tissues in *P. massoniana* exhibited high levels of expression for the SCL family members PmGRAS11, PmGRAS10, PmGRAS8 and PmGRAS15, revealing that these genes may mediate the differentiation and maintenance of meristem cells. Members of the HAM subfamily are mostly expressed in meristem tissues; for instance, they organize the development of the stem apical meristem and axillary meristem in *Capsicum annuum* [58]. PmGRAS10 and PmGRAS20, members of the HAM subfamily, have been shown to be strongly expressed in *P. massoniana* stems and vascular meristems, suggesting that they are essential for the growth of stems and vascular tissues. DELLA members such as HvGRAS18 and HvGRAS60 are expressed in all of the meristematic tissues of barley [59], demonstrating the importance of DELLA genes in plant development [60]. The DELLA subfamily members *PmGRAS9 and PmGRAS17*, stably expressed in eight different tissue sites, are crucial for the growth and expansion of plant tissue in *P. massoniana*.

### 3.4. Subcellular Localization and Transcriptional Activation Analysis of the DELLA Subfamily

The DELLA subfamily is involved in both plant development and stress response. The localization of the genes affects how they function, and subcellular localization provides a framework for understanding gene function. Transient transformation studies have further showed that both PmGRAS9 and PmGRAS17 of the DELLA subfamily are localization factors. This is consistent with our assumption that most PmGRASs are located in the nucleus, with relatively few in the chloroplast.

Members of the DELLA subfamily have been shown to interact with other genes. This subfamily’s IDPs and the highly variable n termini, and the DELLA motif unique to it confer functional properties on the protein as an activator that may block or enhance the activity of interacting partners [61]. For instance, GA promotes the degradation of PIF3 and PIF4 initiated by DELLA, allowing control of gene expression and plant growth in response to light signals [62]. And DELLAs regulate jasmonic acid (JA) by directly interacting with the JAZ1 protein (JA signaling) [63]. Therefore, we chose PmGRAS9 to confirm its transcriptional activation in yeast because it responded significantly to all treatments. By observing the growth status of yeast cells containing the pGBKT7–PmGRAS9 fusion vector on nutrient-deficient medium, it was possible to determine the transcriptional activation activity of these cells. This suggests that these cells can activate the expression of downstream reporter genes, which provides a foundation for studying the regulatory network linked to GRAS proteins.

## 4. Materials and Methods

### 4.1. Plant Materials and Different Treatments

Two-year-old *P. massoniana* seedlings were obtained from the State Key Laboratory of Tree Genetics and Breeding. Seedlings were planted into pots containing a soil mixture (peat:perlite:vermiculite, 3:1:1 (*v*/*v*) at 24 °C with 16 h light and 8 h dark photoperiod. Seedlings with consistent growth status were used in subsequent experiments. We took eight distinct tissues from these seedlings, including terminal buds, young needles, old needles, young stems, old stems, xylem, phloem, and roots to examine the expression level of PmGRASs. Three seedlings with the same growth status were selected as three biological replicates. Additionally, we gave *P. massoniana* seedlings eight treatments: 15% polyethylene glycol (PEG6000), 1 mM salicylic acid (SA), 10 mM methyl jasmonate (MeJA), 50 mM ethylene glycol (ETH), 100 mM abscisic acid (ABA), mechanical damage, 10 mM indole-3-acetic acid (IAA), and 2 mM gibberellin (GA3) treatments. The upper portion of the needles were clipped during the mechanical damage treatment, and spray treatments were used for the remaining treatments. Untreated seedlings served as the controls. Three uniformly developing seedlings were used to represent three biological replicates for each treatment. Every 0 h, 3 h, 6 h, 12 h, 24 h, and 48 h, leaf samples were collected, promptly frozen in liquid nitrogen, and then kept at −80 °C. Control samples were those taken at 0 h without any treatment. 

### 4.2. Identification and Bioinformatics Analysis of GRAS Genes in P. massoniana

The TAIR database (https://www.Arabidopsis.org/, accessed on 6 March 2023) was used to download the *A. thaliana* GRAS protein sequences. The CO_2_ stress transcriptome (PRJNA561037), the drought stress transcriptome (PRJNA595650), and the young branch transcriptome (PRJNA655997) were previously identified as sources of *P. massoniana* transcriptome data. The GRAS gene data were screened using the Markov model (HMM) from the three transcriptomes. Pfam (http://pfam.xfam.org/, accessed on 10 March 2023) and CD-search (https://www.ncbi.nlm.nih.gov/cdd/, accessed on 10 March 2023) were used to search for protein sequences in *P. massoniana* that contain the GRAS structural domain (PF03514.13). Finally, sequences with a complete GRAS structural domain were chosen, and those that shared more than 97% of their similarities with sequences in other databases were eliminated. The physicochemical characteristics of the GRAS protein sequences of *P. massoniana* were examined using the online ExPASy ProtParam tool (https://web.usingexpasy.org/protparam/, accessed on 11 March 2022). CELLO (http://cello.life.nctu.edu.tw/, accessed on 11 March 2022) and PSORT (https://psort.hgc.jp/, accessed on 13 March 2022) were used to predict and assess the subcellular localization of the PmGRAS proteins.

### 4.3. Phylogenetic Analyses and Classifications

Multiple comparisons of the protein sequences from *P. massoniana*, *A. thaliana*, *O. sativa*, and *Populus* were carried out using the ClustalW algorithm in MEGA7 [64], and phylogenetic trees based on the neighbor joining method (NJ) method with 1000 replicates of bootstrap tests. The web program EvolView was then used to alter the phylogenetic tree (https://www.evolgenius.info/evolview/#login, accessed on 16 March 2023) graphically. The phylogenetic tree and conserved domain assemblies were mapped using TBtools [65], and the conserved motifs of GRAS protein sequences were examined using the web software MEME (http://meme-suite.org/tools/meme, accessed on 25 March 2023) with the number of motifs set to 10.

### 4.4. Subcellular Localization and Transcriptional Activity of Candidates

The DELLA subfamily’s PmGRAS9 and PmGRAS17 were chosen for transient expression tests. The primers used to build the vector are described in Table S6 and the coding DNA sequence (CDS) region was inserted into the pBI121-GFP expression vector. For information on gene cloning and vector construction refer to [66]. Green fluorescent protein (GFP)-containing transient expression vectors (35S::PmGRAS9-GFP and 35S::PmGRAS17-GFP) were inserted into the leaves of *N. benthamiana*. The fluorescent signals were seen using a Zeiss LSM 710 confocal microscope (Jena, Germany). Additionally, we included CDS in the pGBKT7 vector. The fusion vector was used to transform the AH109 yeast strain, which was subsequently grown for 48 h at 28 °C on SD/-Trp, SD/-Trop His Ade, and SD/-Trep His Ade (CX11922, Coolaber, Beijing, China) with X-gal selective media.

### 4.5. Transcriptional Pattern Analysis

Transcriptome sequencing data (RNA-seq) obtained following drought treatment were used to quantify the expression levels of GRASs in *P. massoniana*. The information on *P. massoniana* drought treatment for Illumina RNA-seq is displayed below. *P. massoniana* seedlings were sent to Nanjing Forestry University’s greenhouse for three months of acclimatization after being obtained from the *P. massoniana* National Forest Seed Base in Duyun, Guizhou Province, China. For the next studies, 120 sound horsetail pine seedlings of the same height were chosen. The gravimetrical method was used to regulate the soil’s water content. The drought stress lasted for 60 days, and we set four gradients: normal water treatment (CK), mild (T1), moderate (T2), and severe (T3). Each field capacity was set to CK (80 ± 5%), T1 (65 ± 5%), T2 (50 ± 5%), and T3 (35 ± 5%). Each gradient had 10 samples repeated three times. The indoor temperature was controlled at 15–22 °C and the humidity was about 75%. All seedlings were grown under a 16 h light culture (35,000 lx light intensity) and an 8 h dark culture. Using TBtools software, heat maps of particular PmGRASs were made and then examined at the line scale based on log2 values (FPKM + 0.01). Color scales show relative expression levels based on log2 fold change scale values in various gradients.

### 4.6. RNA Extraction and qRT-PCR Analysis

Total RNA was extracted using the RNAprep Pure Kit (Vazyme Biotechnology Co., Ltd., Nanjing, Jiangsu, China), and RNA integrity was estimated by 1.2% agarose gel electrophoresis (Figure S4). First strand cDNA was created using the One-step gDNA Removal and cDNA Synthesis Kit (Vazyme), quantitative real-time reverse transcription PCR (qRT-PCR) primers (Table S6) were designed using Primer 5.0, and SYBR Green reagent was used to detect the target sequences. Each PCR mix (10 µL) contained 1 µL of diluted cDNA (20× dilution), 5 µL of SYBR Green Real-time PCR Master Mix, 0.4 µL of each primer (10 µM), and 3.2 µL of ddH_2_O. PCR quality was estimated from the melting point curve (Figure S5). Among these, 96-Well 0.2 mL Semi Skirt PCR Plates and other experimental consumables were obtained from NEST Biotechnology Co., Ltd. (Wuxi, China). TUA (α-tubulin) [67] was used as an internal control gene. Three biological replicates and three technical replicates were performed. Quantification was performed using the comparative cycle threshold (Ct), and gene expression was calculated using the 2^−∆∆Ct^ method [68].

### 4.7. Statistical Analysis

All experimental data were obtained from at least three replicates, and statistical analysis was performed with Student’s *t*-test. In all experiments, significant differences in the data were evaluated by one-way ANOVA. * *p* < 0.05, and ** *p* < 0.01.

## 5. Conclusions

In this study, we identified 21 GRASs from three transcriptomes and performed their identification and classification as well as various bioinformatics analyses. These genes were divided into nine groups, and PmGRASs from various subfamilies have distinct compositions of motif. Members of the same subfamily share comparable motif compositions, suggesting that they may perform similar roles. The family has a complex evolutionary history in *P. massoniana*, as evidenced by the gene structures and motif compositions of these PmGRASs. The expression of the eight PmGRASs we chose under various conditions demonstrated that they might be important in various stress reactions and serve several purposes at various phases of plant growth and development. Genes from the HAM subfamily and the DELLA subfamily were more responsive to the majority of the treatments, indicating that they may have highly complicated biological functions connected to growth and development as well as stress. These findings contribute to our understanding of the function of PmGRASs, provide a theoretical basis for the study of GRAS TFs, and offer a potential strategy for conducting breeding in *P. massoniana*.

## Data Availability

The data presented in this study are available in Supplementary Materials.

## Supplementary Materials

The following supporting information can be downloaded at: https://www.mdpi.com/article/10.3390/ijms241310690/s1.
