# Supplementary material for: Transcriptome-Wide Identification of the GRAS Transcription Factor Family in Pinus massoniana and Its Role in Regulating Development and Stress Response
Source: Int J Mol Sci. 2023 Jun 27;24(13):10690. doi: 10.3390/ijms241310690 (PMC10341488; doi:10.3390/ijms241310690)
Supplement: Supplementary file 1 [file ijms-24-10690-s001.zip › Supplementary Materials.pdf]

Supplementary Materials

Figures:

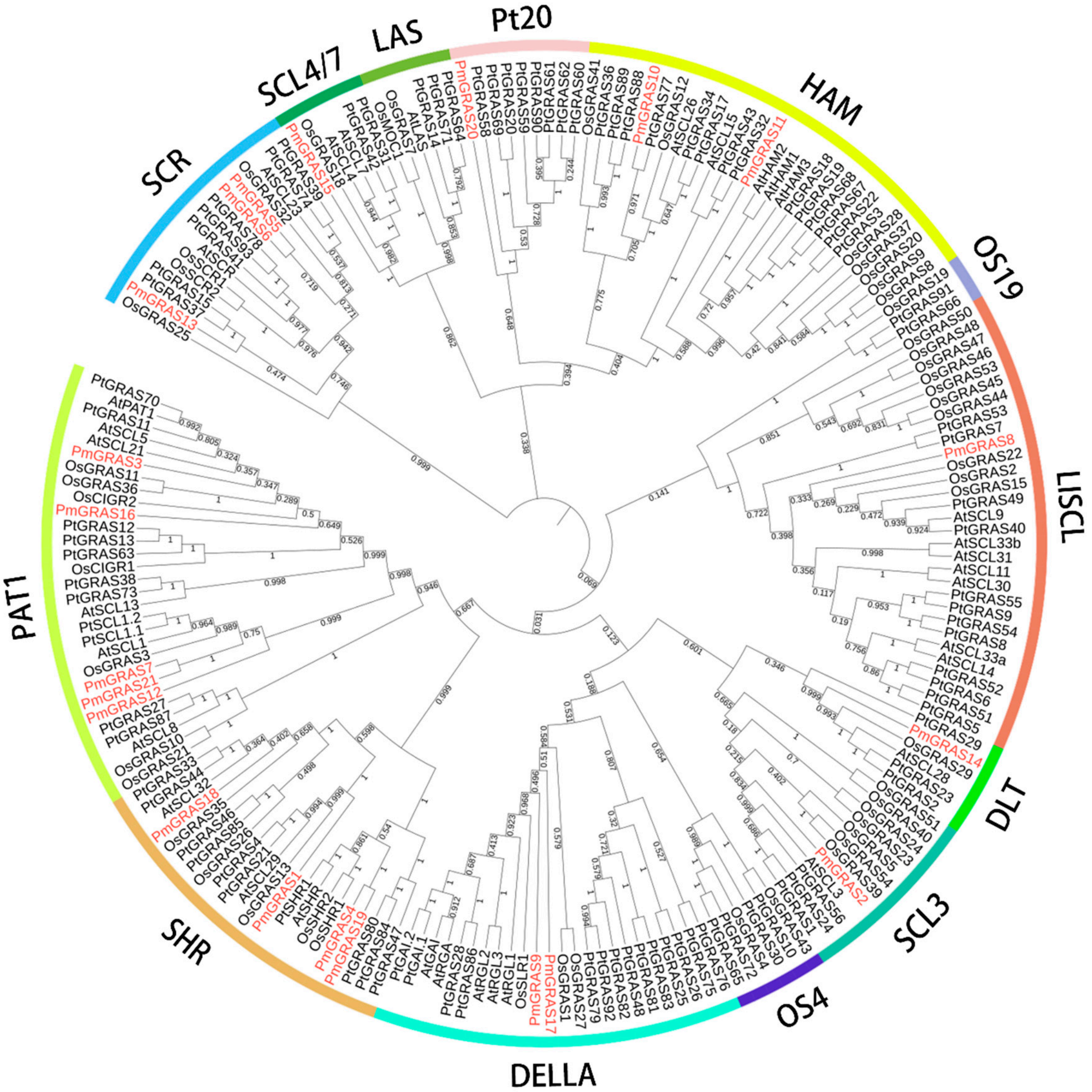

Supplementary Figure S1. The *P. massoniana*, *Populus*, *A.thalinana* and *Oryza sativa* phylogenetic tree, created using MEGA7. The prefixes for the GRAS proteins from *P. massoniana*, *Populus*, *Arabidopsis*, and *Oryza sativa* are "Pm," "Pt," "At," and "Os," respectively.

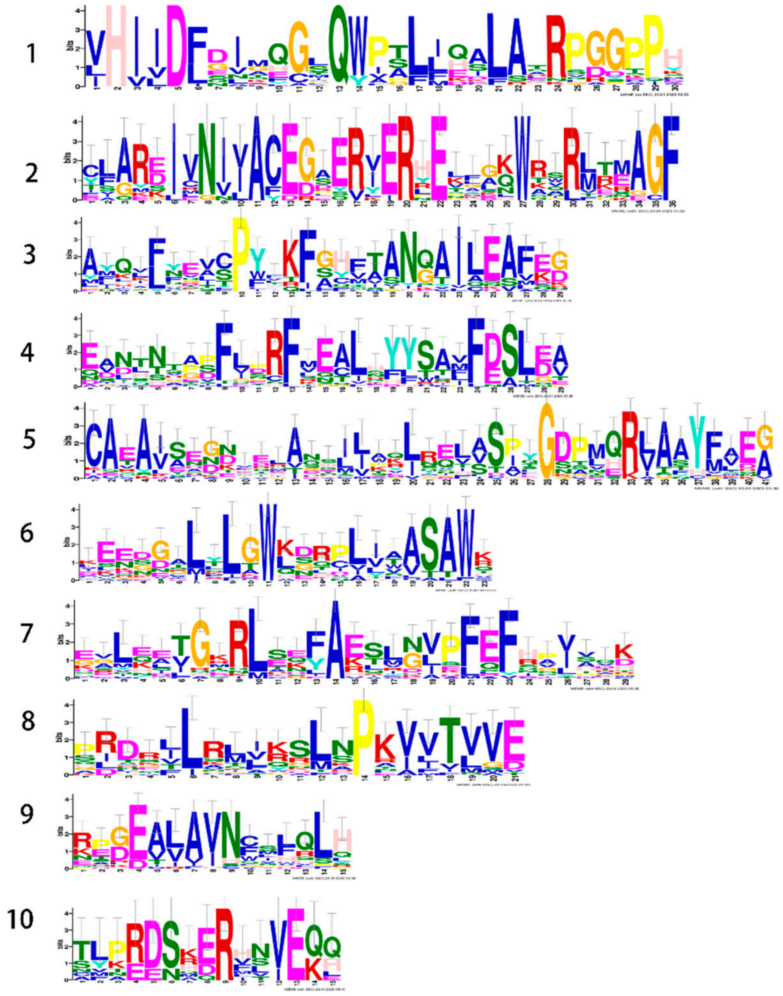

Supplementary Figure S2. 10 site logo depicting the PmGRASs pattern. Each stack of symbols in the logo represents a point in the sequence. The height of the symbols within the stack shows the relative frequency of each amino acid at that position, while the overall height of the stack indicates the sequence conservation at that place.

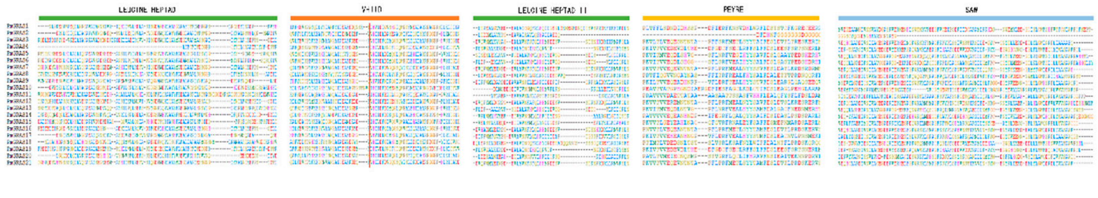

Supplementary Figure S3. The GRAS domains of the 9 evolutionary subfamilies of the *P. massoniana* GRAS protein family have been multiple sequence aligned. The LHR I, VHIID, LHR II, PFYRE, and SAW areas are shown in the scheme at the top as being in the GRAS domain.

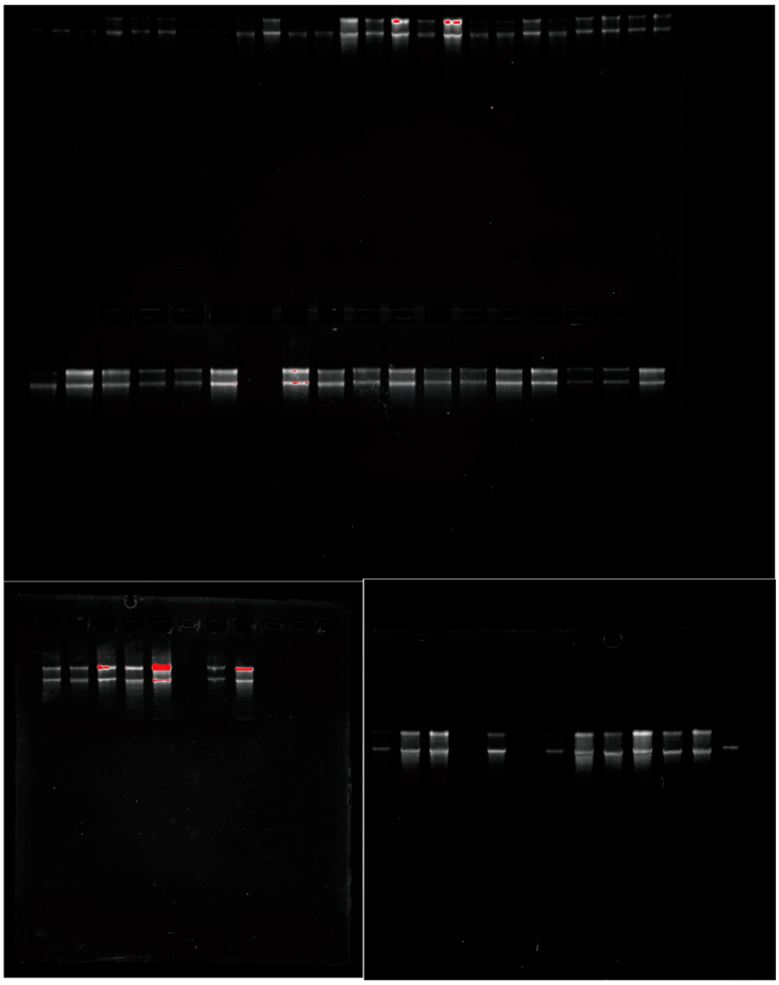

Supplementary Figure S4. Agarose electrophoresis of RNA isolated *P. massoniana*

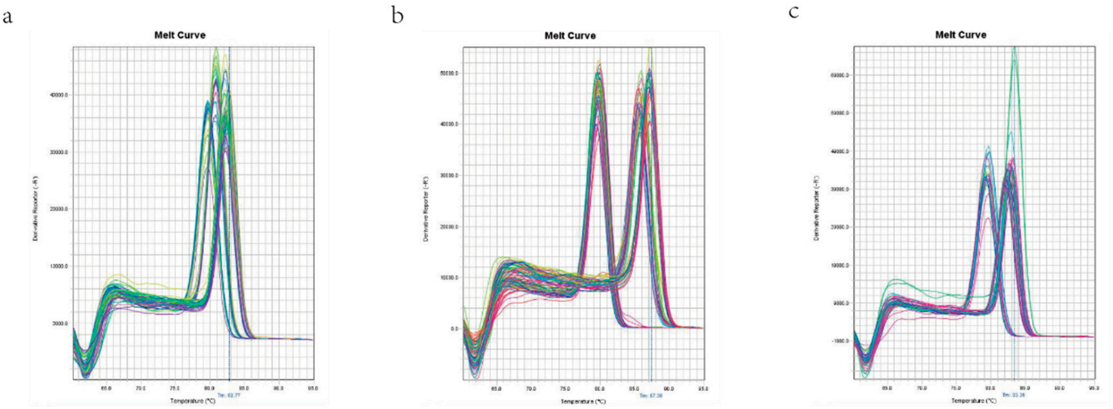

Supplementary Figure S5. Melt curve analysis. (a) Melting curves of *TUA*, *PmGRAS2*, *PmGRAS7* and *PmGRAS8*. (b) Melting curves of *PmGRAS9*, *PmGRAS10* and *PmGRAS11*. (c) Melting curves of *PmGRAS15* and *PmGRAS17*.
